# Perspectives From the Foot and Ankle Department at an Academic Orthopedic Hospital During the Surge Phase of the COVID-19 Pandemic in New York City

**DOI:** 10.1177/1071100720930003

**Published:** 2020-05-25

**Authors:** Jonathan Day, Aoife MacMahon, Matthew M. Roberts, Mark C. Drakos, Anne H. Johnson, David S. Levine, Martin J. O’Malley, Elizabeth A. Cody, Steve B. Behrens, Jonathan T. Deland, Constantine A. Demetracopoulos, Andrew J. Elliot, Scott J. Ellis

**Affiliations:** 1 Hospital for Special Surgery, New York, NY, USA; 2 Weill Cornell Medical College, New York, NY, USA

## Background

Cases of the severe acute respiratory syndrome coronavirus 2 (SARS-CoV-2) virus (COVID-19) first emerged in Wuhan, China, in December 2019.^6^ Since then, the virus has spread globally at a rapid pace. The first case in New York City was reported on March 1, 2020,^8^ and the World Health Organization (WHO) declared a pandemic on March 11, 2020.^9^ New York City has rapidly become the epicenter of the pandemic, with 169 960 confirmed cases and 18 399 deaths as of May 1, 2020.^3^ Hospitals across the city have made a number of changes to accommodate the influx of patients, including cancelling elective procedures, increasing bed capacity, restructuring the workforce, and ensuring a supply of personal protective equipment (PPE). Here, we aim to describe our experience in adapting to the COVID-19 pandemic as a department consisting of 10 foot and ankle fellowship-trained surgeons with up to 28 years of individual experience in an academic orthopedic hospital.

## Immediate Response

On March 17, 2020, a hospital-wide policy was implemented to suspend all elective surgeries in conjunction with surrounding hospitals, including New York Presbyterian’s Weill Cornell Medicine campus (NYP Cornell). This included all elective cases at our main campus and 2 ambulatory surgery centers in Manhattan, as well as at satellite campuses in Stamford, Connecticut, and Long Island. Within days, all service lines, including our Foot and Ankle Department, promptly notified patients who were scheduled for upcoming clinic visits and converted these visits to either telehealth appointments or limited in-person visits. Based on guidelines set forth by the American College of Surgeons COVID-19 Triage Guidelines for Orthopaedic Care,^1^ a list of essential procedures was determined (Table 1), with no additional essential surgeries defined specifically for foot and ankle. Examples of foot and ankle conditions that required immediate surgical treatment included ruptured Achilles tendons, ankle fractures, compartment syndrome, infections, vascular and/or neurologic injuries, and malignancies requiring immediate attention. These guidelines, in addition to other hospital-wide guidelines, were readily accessible to all Hospital for Special Surgery (HSS) employees on our online HSS COVID-19 Preparedness application.

**Table 1.** Essential Foot and Ankle Procedures.

Procedure
Chronic or acute infection requiring surgical intervention
Surgical wound requiring surgical intervention
Compartment syndrome
Dislocation of native or prosthetic joint requiring reduction in the operating room
Malignant or benign tumors with impending fracture or neurovascular compromise
Fractures (periprosthetic or native bone)
Loose body in the joint or displaced cartilage causing locked joint
Injury to the lower extremity where the patient has an inability to bear weight or walk
Any vascular or neurologic injury
Tendon injury
Injury where prompt intervention significantly improves outcomes

## Patient Care

In the Foot and Ankle Department, our goals were to provide existing patients with the follow-up they needed and to care for new patients in an effective and timely manner. Starting on March 17, 2020, in-person clinical visits were limited to new patients being evaluated for essential procedures or first postoperative visits for returning patients. This included patients presenting for cast changes and pin removals. All other new patient and follow-up appointments, including preoperative clearance, were converted to online telehealth visits using the Zoom platform, integrated through the Epic (Epic Systems) electronic health record.

With the decrease in patient volume and the aim to minimize use of clinical space, clinic visits at our main hospital were transitioned from our department office building to a centralized location. This helped reduce the numbers of staff required. Patients who required emergent surgery were triaged and placed in the next available operating room slot. Imaging, including x-rays, magnetic resonance imaging, and computed tomography, remained available onsite for patients who required it. For patients consulting via telemedicine, patients have been successfully referred for imaging to a host of outside centers in nonhospital settings, closer to their homes. These images were subsequently transferred to our foot and ankle providers as well as radiologists and most commonly were either uploaded directly by the patient into our picture archiving and communications system (PACS; Sectra) or into the electronic medical system by our office staff. In some cases, surgeons were able to access physician portals at various radiology facilities to gain access to relevant images. In addition, physical therapy was continued for all recent surgical patients who required it, with a transition to telehealth visits for all eligible patients both at our institution and others. Many physical therapy centers at more remote locations outside New York City remained open but saw limited numbers of patients.

To optimize staff and supply allocation, all orthopedic surgeries were consolidated into 1 main floor with 5 active running operating rooms (ORs). In the OR, in addition to standard scrubbing and sterile gowning procedures, PPE has been used with precautions assuming that any patient may be COVID positive, including use of N95 respirator masks, in accordance with Centers for Disease Control and Prevention (CDC) guidelines.^2^ For foot and ankle procedures, we have avoided general anesthesia to the greatest extent possible to reduce the risk of virus aerosolization.

In addition, an Orthopedic Triage Center (OTC) was established on the first floor of our main hospital in the location where we normally perform foot/ankle and hand surgery at our institution. The goals of this were to relieve the patient load in the emergency departments at New York Presbyterian Hospital and other hospitals in New York City, as well as to minimize these patients’ exposure to COVID-positive patients while continuing to deliver urgent orthopedic care. The OTC provided care for patients with orthopedic conditions only; was open 24 hours a day, 7 days a week; and was staffed by registered nurses, physician assistants, residents, and fellows with attending physician oversight. Patients could be brought to the OTC directly by emergency medical services, through referral by a provider from our institution, or directly through the NYP Cornell Emergency Department.

To provide support for the increasing patient load at hospitals across New York City, much of our main hospital was restructured and repurposed to accept transfers of both COVID-negative and COVID-positive patients, with an expanded capacity of 170 medical surgical beds and 30 critical care beds. Of our 5 inpatient floors, 1 floor was dedicated to noncritical COVID-positive patients, and an additional floor was dedicated to telemetry monitoring of COVID-positive patients. A postanesthesia care unit (PACU) and a floor of 9 operating rooms were repurposed as negative-pressure rooms for the care of critical COVID-positive patients on ventilators. Each OR was modified to accommodate 2 ventilated COVID patients. The PACU was reconstructed to have a central, protected nursing station and multiple beds that could accommodate COVID patients. These beds were not isolated from one another. One floor was used for COVID-negative medical surgical patients, a step-down unit (SDU) was used for COVID-negative patients, and an orthopedic special care unit (OSCU) was utilized as an intensive care unit (ICU) for critical COVID-negative patients on ventilators. Two floors were maintained for orthopedic inpatients. Patient care teams were each composed of a medical attending with assistance from an orthopedic fellow and/or volunteer orthopedic faculty, orthopedic residents, physician assistants, and nurses. Our foot and ankle surgeons and fellows have been active members of these patient care teams. Our 76 anesthesiologists, including 6 with critical care training and 20 others with critical care experience, have been integral in caring for patients on ventilators.

To provide further support to NYP Cornell, our institution reallocated supplies where possible to our neighboring institution. With the shutdown of all but 5 ORs at our main hospital, as well as our 2 ambulatory surgery centers with a total of 6 ORs, 21 ventilators and 50 anesthesia machines were made available, many of which were sent to NYP Cornell. In addition, our institution reallocated 36 000 N95 respirators and 576 goggles to our neighboring institution.

## Employee and Patient Safety

Our hospital has implemented several policies to keep our employees and patients safe while still fulfilling our primary goal of delivering timely and effective orthopedic care. Transitioning to a telehealth system for all nonurgent clinic visits has helped ensure that we are providing care to patients while simultaneously promoting social distancing. To further facilitate a safer work environment, scheduling offices and physicians’ offices, which ordinarily have up to 3 or 4 staff members working at a time in each office, have been reduced to allow only 1 employee present in the office each day. In many cases, the providers were the only team members in the hospital with the entire staff working remotely. Nonessential staff, including research personnel and office staff, have been permitted to work from home with remote access to continue their responsibilities. Those staff that did not have work to perform were still paid at 80% of normal salary with benefits and have had their jobs held until we are able to return to more normal operations.

Similarly, by consolidating all in-person patient visits on our main campus to 1 centralized location, we have been able to accomplish several important goals. First, this minimizes the use of resources, particularly PPE, and increases the capacity in our facilities to better provide support to NYP Cornell. Second, this consolidated model has allowed us to implement a rotational schedule of attending surgeons and essential personnel. This, in turn, has allowed us to provide essential in-person care to existing patients and new patients with urgent issues while limiting exposure to staff and practitioners by decreasing the number of physicians and ancillary staff onsite, with only 1 or 2 physicians per subspecialty on the main campus per day. In addition, this has helped us to maintain standardized COVID-19 screening and protocols, and we have been able to control the number of patients seen at any given time and also limit the number of patients present in the waiting room areas.

Several measures have been implemented to minimize COVID-19 exposure among staff and patients. All staff are required to wear surgical masks and eye protection for all clinical interactions regardless of patient COVID status. Additional precautions are taken during procedures that could create aerosolized particles or in patients who are known to be COVID-positive. The clinic space has been set up to maintain social distancing, including appropriate spacing of seats in waiting rooms, time between patients visits, and a thorough cleaning plan. Moreover, all clinic patients are screened for COVID-19 with a 3-question phone screen prior to arrival, which includes questions regarding COVID status and relevant clinical symptoms, as well as an in-person screen on arrival that includes a temperature check. If there is concern for COVID-19 based on an initial phone screen, patients are directed to the emergency department. If patients screen positive at the clinic visit, they are masked upon arrival and immediately escorted to a designated room by a licensed practical nurse (LPN) in appropriate PPE. Once the patient visit is complete, the LPN escorts the patient out of the building, and examination rooms are cleaned before the next patient is seen. Follow-up care is coordinated remotely by physician office staff.

Testing for COVID-19 was initially performed by sending nasopharyngeal swab specimens to our neighboring institution, Memorial Sloan Kettering Cancer Center, for results. On April 13, 2020, our institution was able to start performing in-house polymerase chain reaction (PCR) testing. Patients eligible for testing included all admitted patients, all surgical inpatients, all patients presenting to the OTC, and all transferred patients, unless the patient had a negative test in the past 48 hours or a prior positive test. As of May 1, 2020, in-house COVID-19 serologic testing was made available to all staff, with a tiered approach to testing based on frontline work and history of symptoms or previously confirmed COVID-19.

## Sustainability of Our Approach

Since the start of our hospital-wide response to the coronavirus, we have successfully transitioned to a telehealth and triaging model to continue delivering care to patients with foot and ankle problems. Over the month of April, our Foot and Ankle Department alone had over 600 telehealth appointments and 200 in-person appointments. During the surge phase of the COVID-19 pandemic, the hospital as a whole operated at a 10% caseload compared to normal. To date, we have been able to perform all nonelective surgical cases without any significant delays. We continue to provide orthopedic care at our satellite locations in New York, New Jersey, and Connecticut. In regards to research, the hospital as a whole has leveraged its resources toward the community response to combatting COVID-19, including clinical trials and a nationwide convalescent plasma program approved by the US Food and Drug Administration.^5,7^ With these ongoing efforts, we remain cognizant of the continued course of this pandemic and therefore have implemented strategies to sustain our approach and our obligation to our patients and health care workers.

As an academic teaching hospital, we have emphasized continuing resident and fellow education. Within the Foot and Ankle Department, residents and fellows continue to assist with orthopedic cases and seeing orthopedic patients in urgent care centers, as well as cross-covering to provide orthopedic and nonorthopedic inpatient care. However, fellows and residents are not currently assigned to cover nonurgent clinics or participate in telemedicine visits. All academic conferences, imaging didactics, and research meetings have been transitioned to online conferences and webinars. Many of these lectures are shared across the country through a fellowship collaborative partnership with the American Orthopaedic Foot & Ankle Society (AOFAS) in an effort to continue education in foot and ankle surgery.

A huge asset to the swift transition and sustained momentum of our approach was our transparency. Both hospital-wide and within our own service, it has been a priority to keep all lines of communication open to ensure the safety of each employee. These include daily email correspondences from the hospital’s leadership as well as live webinars open to all employees as an open forum for discussion.^4^ Our institution assembled a COVID-19 Task Force as well as a Hospital for Special Surgery COVID-19 Preparedness online application to efficiently centralize and disseminate guidelines and updates. Within our department, our service line managers and faculty continue to send weekly emails to maintain correspondence of the latest updates. These open lines of communication have been crucial in sustaining our efforts against the constantly evolving circumstances of this crisis.

Through changing the way we deliver foot and ankle care and by repurposing the roles of our employees and our institution, we have adapted strategies to continue delivering care to our patients. As we transition toward a “new normal,” our goals are to gradually progress toward normal operations while keeping our patients and employees safe. This includes providing surgical care to an expanding category of urgent and priority orthopedic conditions, as well as increasing outpatient visits. As of May 4, 2020, each physician was assigned 1 day of outpatient clinic time per week, limited to a maximum of 2 examination rooms and 24 patients, to ensure adequate social distancing and cleaning between visits. A coverage schedule will continue to accommodate emergent and urgent patients. With these gradual steps, we hope to emerge from this pandemic stronger and ready to adapt to the everchanging needs of our community.

## Supplemental Material

FAI930003_disclosures – Supplemental material for Perspectives From the Foot and Ankle Department at an Academic Orthopedic Hospital During the Surge Phase of the COVID-19 Pandemic in New York City Click here for additional data file. Supplemental material, FAI930003_disclosures for Perspectives From the Foot and Ankle Department at an Academic Orthopedic Hospital During the Surge Phase of the COVID-19 Pandemic in New York City by Jonathan Day, Aoife MacMahon, Matthew M. Roberts, Mark C. Drakos, Anne H. Johnson, David S. Levine, Martin J. O’Malley, Elizabeth A. Cody, Steve B. Behrens, Jonathan T. Deland, Constantine A. Demetracopoulos, Andrew J. Elliot and Scott J. Ellis in Foot & Ankle International
